# Development of an *in vitro* co-culture model to mimic the human intestine in healthy and diseased state

**DOI:** 10.1016/j.tiv.2017.08.011

**Published:** 2017-12

**Authors:** Angela A.M. Kämpfer, Patricia Urbán, Sabrina Gioria, Nilesh Kanase, Vicki Stone, Agnieszka Kinsner-Ovaskainen

**Affiliations:** a European Commission Joint Research Centre, Directorate F - Health, Consumers and Reference Materials, Via E. Fermi 2749, 21027 Ispra, VA, Italy; b Nano-Safety Research Group, School of Engineering and Physical Sciences, Heriot-Watt University, Edinburgh EH14 4AS, United Kingdom

**Keywords:** Co-culture, Intestine, Inflammation, Inflammatory bowel disease, Macrophages

## Abstract

The intestine forms the largest interface between the environment and the human organism. Its integrity and functioning are crucial for the uptake of nutrients while preventing access of harmful antigens. Inflammatory conditions can significantly change the normal functioning of the intestine. *In vitro* models that adequately reproduce both healthy and inflamed intestinal tissue could provide a useful tool for studying the mechanisms of intestinal inflammation and investigating new therapeutic drugs.

We established a co-culture of Caco-2 and PMA-differentiated THP-1 cells that mimics the intestine in healthy and controlled inflamed states. In homoeostatic conditions without stimulation, Caco-2 and THP-1 cells were co-cultured for 48 h without affecting the barrier integrity and with no increase in the release of cytokines, nitric oxide or lactate dehydrogenase. To simulate the inflamed intestine, the Caco-2 barrier was primed with IFN-γ and THP-1 cells were pre-stimulated with LPS and IFN-γ. In these conditions a significant but temporary reduction in barrier integrity was measured, and large concentrations of pro-inflammatory cytokines and cytotoxicity markers detected.

With its ability to feature numerous hallmarks of intestinal inflammation the presented co-culture model of epithelial cells and macrophages offers a unique possibility to study exposure effects in relation to the health status of the intestine.

## Highlights

• A novel, tunable co-culture model of Caco-2 and THP-1 cells was established.
• The THP-1 differentiation protocol is crucial for a stable co-culture with Caco-2.
• Synergistic effects of TNF-α and IFN-γ were key to induce inflammation *in vitro*.
• The inflamed co-culture shows barrier disruption, cytokine release and cytotoxicity.
• Downregulation of inflammation is prevented by pretreatment of cells with cytokines.

## 1 Introduction

The intestine is the main site for digestion, nutrient uptake and water regulation (Kvietys and Granger, 2010). Its functions are balanced between the uptake of nutrients across the intestinal epithelial barrier and prevention of intrusion of harmful antigens (Clayburgh et al., 2004). To fulfil this ambivalent role, intestinal epithelial cells (IECs) and the gut-associated lymphoid tissue (GALT) have established a finely-tuned cohabitation. Firmly connected by tight junctions (TJs), the IECs form a semi-permeable barrier that restricts the translocation of luminal material. In the underlying lamina propria, non-inflammatory intestinal macrophages contribute to the protection from invading pathogens by active phagocytosis (Smythies et al., 2005).

For decades, *in vitro* models of the intestine have been used to study the pharmacological and toxicological effects, as well as the bio-availability of substances (Sambruy et al., 2001) and materials (Desai et al., 1997). Although the development of primary cell- (Castellanos-Gonzalez et al., 2013) and stem cell-derived (Chopra et al., 2010) models has advanced recently, the application of immortalised cell lines like Caco-2 remains favoured due to their easy accessibility, handling, and maintenance.

Best known for their spontaneous differentiation from a colonic to a small intestinal-like phenotype (Pinto et al., 1983), Caco-2 cells offer transport and permeability characteristics similar to human intestinal tissue (Lennernas et al., 1996, Rubas et al., 1993). The application of Caco-2 cells has generated countless valuable results regarding the pharmacokinetics and toxicological impacts of compounds. Cell monocultures are, however, not capable of mimicking the complex structure defining the intestine. Whereas they are valued to study specific endpoints, *e.g.* cytotoxicity, their ability to predict downstream impacts in relation to the biokinetics and metabolism of substances is limited (Duell et al., 2011, Lilienblum et al., 2008). Therefore, the development of more sophisticated intestinal co-culture models combining different cell types is favoured. Depending on the desired application, advancements addressed an improved representation of the intestinal barrier (Wikman-Larhed and Artursson, 1995, Schimpel et al., 2014, Georgantzopoulou et al., 2016) or the integration of immune cells (Bisping et al., 2001, Leonard et al., 2010, Susewind et al., 2016).

The application of these increasingly complex cell models generated new insights regarding the regulation of intestinal homeostasis (Parlesak et al., 2004, Nathens et al., 1995) and confirmed the influence of immune cells on IEC responsiveness to stressors (Wottrich et al., 2004, Susewind et al., 2016, Moyes et al., 2010). Most of these models, however, were established using primary cells (Leonard et al., 2010, Bisping et al., 2001), which can negatively affect the reproducibility and the inter-laboratory comparability of the results (Corazza and Wade, 2010), or using cell lines of non-human origin (Tanoue et al., 2008). Others were characterised by a spontaneous disruption of the epithelial barrier in the co-culture and uncontrolled inflammation-like processes (Watanabe et al., 2004, Satsu et al., 2006, Kanzato et al., 2001, Moyes et al., 2010).

Here we present an *in vitro* co-culture model of cell line-derived IECs (differentiated Caco-2 cells) and macrophages (differentiated THP-1 cells) that can be manipulated to mimic the intestine in either homeostatic or inflamed states. Cell lines of human origin were favoured to maximise the model's applicability to a human exposure scenario, while standard cell lines were used to make the model accessible throughout other laboratories.

## 2 Materials & methods

### 2.1 Materials

Foetal bovine serum (FBS), phosphate buffered saline (PBS), minimum essential medium (MEM), RPMI medium, sodium pyruvate, Penicillin/Streptomycin, 2-mercaptoethanol, trypsin/ethylenediaminetetraacetic acid (EDTA), l-Glutamine, Phalloidin AlexaFluor488-conjugated (Cat.: A12379), 4′,6-Diamidino-2-Phenylindole (DAPI) (Cat.: D1306), anti-zonula occludens antibody (Cat.: 617300), and AlexaFluor546 (Cat.: A20183) were purchased from Thermo Fisher Scientific (Monza, Lombardy, Italy). d-Glucose, Triton X-100, phorbol 12-myristate 12-acetate (PMA), formaldehyde, Tris-base, Tris-HCl, lithium lactate, *E. coli*-derived lipopolysaccharide (LPS), interferon (IFN)-γ, β-nicotinamide adenine dinucleotide sodium salt (NAD), iodonitrotetrazolium chloride (INT), phenazine methosulfate (PMS), sulfanilamide (SA), *N*-(1-naphthyl)-ethylenediamine (NEDA), sodium nitrite (NaNO_2_), Accutase, EDTA, Tween20, and bovine serum albumin (BSA) were obtained from Sigma-Aldrich (Milan, Lombardy, Italy).

### 2.2 Cell cultures

Caco-2 cells (ACC169, DSMZ; Braunschweig, Lower Saxony, Germany) were cultured in MEM-based cell culture medium (CCM) substituted with 20% heat-inactivated FBS and 1% Penicillin/Streptomycin at 37 °C, 5% CO_2_. For co-cultures, the cells were seeded on transwell inserts (1 μm pore size; Falcon, Sacco S.r.L., Cadorago, Como, Italy) at a density of 1.8E + 5 cells cm^− 2^ and maintained for 18–21 days. On the apical (AP) side, the cells were cultured in MEM, whereas the medium in the basolateral (BL) compartment was changed (Supplementary, Table S1) to RPMI-based THP-1 medium without mercaptoethanol (composition below).

The THP-1 cells (TIB-202, ATCC; *via* Sigma-Aldrich) were cultured in RPMI-based CCM substituted with 10% heat-inactivated FBS, 1% Penicillin/Streptomycin, 1% l-Glutamine, 1 nM sodium pyruvate, 0.7% d-Glucose, and 0.1% mercaptoethanol at 37 °C, 5% CO_2_. For co-culture experiments, THP-1 cells were seeded (3E + 6 cells) in 25 cm^2^ flasks and differentiated with PMA (100 nM). Subsequently, the cells were detached with Accutase, plated on transwell-suitable 12-well plates at a density of 1.8E + 5 cells/well and allowed to re-attach for 1.5 h. Further details are reported in the results section.

Both cell lines were tested for their genetic integrity (DSMZ) and for mycoplasma contamination by qPCR (Minerva Biolabs GmbH; Berlin, Germany). The results showed fully matching STR reference profiles compared to the distributors' profiles and no contamination with non-human genetic material or mycoplasma.

### 2.3 Stable co-culture model mimicking the healthy human intestine

The first aim of the project was to establish a co-culture of Caco-2 and PMA-differentiated THP-1 cells to mimic the human intestine in homeostatic state (hereinafter ‘stable co-culture’). For a co-culture to be qualified as ‘stable’, the following criteria were set:

• Changes in the growth environment can cause a temporary reduction in TEER by ~ 10%. Therefore, the barrier's TEER should decrease ≤ 10% compared to the Caco-2 monoculture control over the first 24 h of co-culture.
• The TEER should re-establish to ≥ 95% after 24 h of co-culture.
• The co-culture should not induce an activation of the macrophages or stimulate the IECs. Therefore, the concentrations of pro-inflammatory cytokines in the BL compartment should not significantly exceed the cytokine levels released by THP-1 monocultures without stimulation.
• To confirm the absence of necrotic cell death the apical LDH content was not to be significantly higher compared to the Caco-2 monoculture.

### 2.4 Inflamed co-culture model

The second aim was to disrupt the stable co-culture through the induction of an inflammation-like response caused by activated THP-1 cells (hereinafter ‘inflamed co-culture’) to mimic the diseased intestine. To obtain comparable results between the two models, the number of variables between them should be kept to a minimum. Additionally, the *in vitro* inflammation was designed to resemble a biological response as closely as possible. Hence, the following requirements were defined for the inflamed model:

• The inflammatory response should be induced through a physiologically relevant stressor, here the endotoxin LPS, which is found on the membrane of Gram-negative bacteria.
• The model should resemble intestinal inflammatory processes as closely as possible. Therefore, no substantial or permanent destruction of the Caco-2 barrier was to occur.
• The system ideally has to recover itself and resolve the inflammation-like process without additional manipulation of the culture.

The following criteria for a co-culture to be classified as ‘inflamed’ were defined:

1. A TEER reduction by at least 20–25% compared to the Caco-2 monoculture should be obtained.
2. A TEER reduction of minimally 20% had to persist for at least 24 h.
3. After 4 h of co-culture, the levels of pro-inflammatory cytokines need to significantly exceed the concentrations recovered in the stable co-culture.
4. Ideally, the TEER should re-establish to > 90% of the Caco-2 monoculture control after 48 h of co-culture.

Co-cultures which did not comply with the above defined criteria were excluded from the result calculations.

### 2.5 Monitoring of barrier integrity by TEER

The TEER was measured using an Ohm-meter (MERSSTX01, Millipore Millicell; Billerica, MA, USA) to assess the barrier development of the Caco-2 cell layer (every 2–3 days of culture), as well as the barrier integrity throughout the co-culture with THP-1 cells (after 4, 18, 24, and 48 h). The electrode was sterilised in 70% ethanol (15 min) and neutralised in PBS and MEM. The results were corrected for the blank and multiplied by the filter size (0.9 cm^2^) to obtain the final results in Ohm per cm^2^ (Ω·cm^2^). Caco-2 monocultures reached the maximum TEER between 12 and 15 days post-seeding with 510 ± 45 Ω·cm^2^ (Fig. S1). Subsequently, the barrier resistance decreased to ~ 396 ± 28 Ω·cm^2^ at day 21. Hereinafter, the TEER results of co-culture experiments are expressed as percentage of the Caco-2 monoculture control values.

### 2.6 Cytokine quantification

#### 2.6.1 Enzyme-Linked Immuno-Sorbent Assay (ELISA)

The release of IL-1β, IL-8, TNF-α, and TGF-β1 was quantified using cell-free supernatants from the BL compartment after 4, 24, or 48 h of co-culture. The ELISA was run as described by Kinsner et al. (2006) using commercially available antibody pairs (R&D Systems, Cat.: DY210, DY201, DY208, DY240; Abingdon, Oxfordshire, UK). Briefly, the primary antibodies were incubated overnight at room temperature (RT) in coating buffer (0.1 M NaHCO_3_ in MilliQ H_2_O) on high protein-binding 96-well plates (Thermo Fisher Scientific). After washing (PBS + 0.05% Tween20) and blocking (3% BSA/PBS) for 1 h at RT, the cytokine standards and supernatant samples were added and incubated for 2 h at RT, followed by incubation for 45 min with biotinylated secondary antibodies. After 30 min incubation with streptavidin-peroxidase (Biotrend Chemikalien; Cologne, NRW, Germany), 100 μL 3,3′,5,5′-Tetramethylbenzidine (Sigma-Aldrich) was added and the reaction was stopped with sulfuric acid (1 M) after 5 (IL-8) or 15 min (TNF-α, TGF-β1, and IL-1β). The absorbance was read spectrophotometrically (Enspire, Perkin Elmer; Milano, Lombardy, Italy) at 450 nm.

#### 2.6.2 Bio-Plex Magpix

The release of TNF-α, IL-4, IL-6, IFN-γ, granulocyte macrophage colony-stimulating factor (GM-CSF), macrophage inflammatory protein (MIP)-1α, and monocyte chemoattractant protein (MCP)-1 were quantified using a magnetic bead-based assay (Bio-Rad Laboratories, Cat.: 171B50-004, -006, -018, -019, -021, -022, -026; Segrate, Milan, Italy) analysed with the Bio-Plex MAGPIX Multiplex Reader (Bio-Rad Laboratories; Hemel Hempstead, Hertfordshire, UK). For the analysis, supernatant samples were taken from the BL compartment after 28 h of co-culture. The master mix was incubated with undiluted supernatant samples from the monoculture and stable co-culture, or 1:2 diluted samples of the inflamed co-cultures for 30 min at RT. Subsequently, the detection antibodies were added and the plate incubated again for 30 min at RT. Eventually, the wells were incubated with streptavidin-PE and incubated for 10 min at RT before the beads were re-suspended in assay buffer and the plate read using a Bio-Plex MAGPIX Multiplex Reader. Blanks and standard curves were included on each plate.

### 2.7 Nitrite detection using the Griess reaction

The presence of nitrite (NO_2_^−^), an indicator of nitric oxide (NO) synthesis, was assessed in supernatant samples collected after 48 h in all three culture models. In 3 steps separated by 30 min incubation time, SA (final concentration 100 μM), H_3_PO_4_ (170 mM), and NEDA (100 μM) were added to each supernatant sample (100 μL). The absorbance of the formed azo dye was measured spectrophotometrically (Enspire, Perkin Elmer) at 548 nm. Before the concentration of NO_2_ˉ was calculated from the calibration curve (0–50 μM NaNO_2_ in MEM or RPMI), the background absorbance was subtracted from the raw data.

### 2.8 Quantification of lactate dehydrogenase (LDH) release

The assay was used to quantify the enzymatic activity of LDH, which is released after cell damage or necrotic cell death. Briefly, 50 μL of 200 mM TRIS, 50 μL of 50 mM lithium lactate, and 50 μL mix of INT, PMS, and NAD at a concentrations of 1.32 mg mL^− 1^, 0.36 mg mL^− 1^ and 3.44 mg mL^− 1^, respectively, were added to a 96-well plate. Subsequently, 50 μL of cell-free supernatant were transferred and incubated for 5 min at RT. The optical density was measured spectrophotometrically (Enspire, Perkin Elmer) at 490 nm. A background control in complete CCM was subtracted from the results. Cells exposed to 0.1% Triton X-100 in PBS for 24 h served as control for 100% cell lysis.

### 2.9 Immunocytochemical staining and analysis

To study the effect of THP-1 cells on the barrier integrity, the Caco-2 cells were stained for nuclei (DAPI), the cytoskeleton (F-actin), and the TJ protein zonula occludens (ZO)-1 after 48 h of co-culture, and analysed by fluorescence microscopy or the IN Cell Analyzer (GE Healthcare; Pittsburgh, PA, USA).

The cells were washed with PBS, fixed in 3.7% formaldehyde (13 min), permeabilised with 1% Triton X-100 in PBS (5 min), and blocked against unspecific binding with 3% BSA/PBS (30 min). Afterwards, the cells were incubated with the anti-ZO-1 primary antibody (5 μg mL^− 1^) for 1.5 h at RT. After washing, the cells were incubated with the secondary antibody labelled with AlexaFluor546 (1:300), Phalloidin-AlexaFluor488 (1:40), and DAPI (1:4000) in 1% BSA/PBS for 45 min at 37 °C to stain the TJs, actin filaments, and nuclei, respectively.

#### 2.9.1 Fluorescence microscope

For analysis, the stained filters were cut from the supports and mounted cells facing up on standard microscopy glass slides. Images were acquired with an Axiovert 200M inverted microscope (Carl Zeiss; Jena, Thuringia, Germany) equipped with ApoTome slide module and AxioVision 4.8 software (Carl Zeiss; Jena, Thuringia, Germany), using 40 ×/1.0 objective lens. The acquisition time was set individually for each channel using the monoculture control and subsequently applied for the analysis of all samples. The channel focus was adjusted manually. A black and white AxioCam MRm (Carl Zeiss; Jena, Thuringia, Germany) was used and pseudocolors were applied after image acquisition. The images were subject to processing using ImageJ (freeware at https://imagej.nih.gov/ij/). All images were treated with the same background subtraction. In case the signal intensity was increased a linear enhancement was applied to the whole file.

#### 2.9.2 IN Cell analyser

For the analysis, stained filters were left in the supports and well plates. The filters were imaged using a 60 × objective. To obtain a higher resolution, images were acquired as a z-stack of three images of 1 μm each, which was converted into a 2D image using the IN Cell Investigator Software (GE Healthcare, Cardiff, Wales, UK).

### 2.10 Lucifer Yellow (LY) assay

To compare the paracellular permeability in the Caco-2 monoculture and the co-culture conditions the passage of LY from the AP to the BL compartment was measured. LY was dissolved in MEM (5 mg mL^− 1^) and added to the AP compartment at the start of the co-culture. From both sides, 100 μL samples were taken immediately after addition of LY and after 4 and 18 h of incubation/co-culture. The fluorescence was measured spectrophotometrically at 485/530 nm (excitation/emission). The dye's apparent permeability (P_app_) was calculated from samples taken after 18 h using the following equation:

(1) $$P_{\mathrm{app}}=\frac{\mathrm{dQ}/\mathrm{dt}}{{C_{0}}^{*}A}$$

with dQ/dt being the permeability rate in μM/s, C_0_ the initial concentration of LY in the AP compartment (5 μM), and A the filter surface area in cm^2^ (0.9 cm^2^). The results are expressed as cm s^− 1^ (Ma et al., 2014).

### 2.11 Statistical analysis

Results were generated from 3 independent experiments (n = 3) with 3 technical replicates unless stated otherwise. The data analysis was performed with Microsoft Excel. The results were illustrated using GraphPad Prism 6 and variations between results were expressed as standard deviation (s.d.). The data were statistically analysed by one-way ANOVA and post-hoc Dunnett's test, unless stated otherwise, using Minitab. A value of p ≤ 0.05 was accepted as statistically significant. No graphical difference was made for p-values ≤ 0.05–0.001.

## 3 Results

### 3.1 PMA-induced differentiation of THP-1 cells

We first investigated the impact of different PMA-differentiation protocols on the behaviour and characteristics of THP-1 cells. The PMA differentiation should result in an increased cellular response to pro-inflammatory stimuli, which was confirmed by comparing the release of IL-1β, IL-8, and TNF-α of PMA-treated THP-1 cells without stimulation (hereinafter ‘unstimulated’) and after 4 h stimulation with 10 ng mL^− 1^ LPS (Fig. S2).

Our initial studies on PMA-treated THP-1 cells used a differentiation protocol of 24 h PMA-pre-treatment plus a 24 h rest period (hereinafter ‘48 h-differentiated’). The 48 h-differentiated THP-1 cells readily released noticeable concentrations of cytokines even without LPS-stimulation. The levels of IL-8 were particularly high with > 4400 pg mL^− 1^ (Fig. S2). Both IL-1β and TNF-α were expressed at lower but detectable concentrations (Fig. S2). The exposure to LPS (10 ng mL^− 1^) significantly enhanced (p ≤ 0.001) the release of all three cytokines to between 2.5- and 12-fold (Fig. S2). Undifferentiated THP-1 cells on the other hand barely responded to the stimulation with LPS (Fig. S3).

**Fig. 1:**
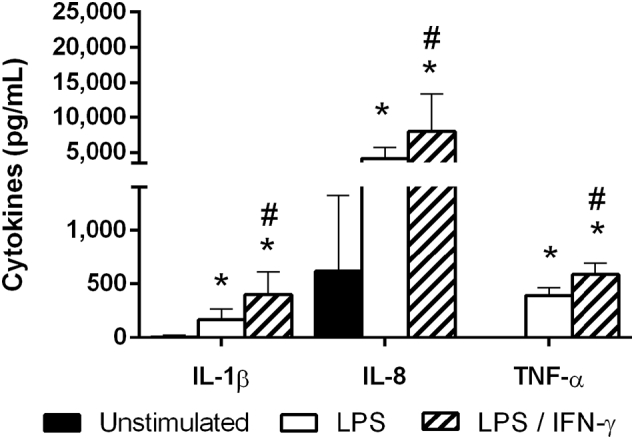
Release of IL-1β, IL-8, and TNF-α by 24 h PMA-differentiated THP-1 cells: without stimulation (black bars) or 4 h stimulation with LPS (white bars) or LPS + IFN-γ (striped bars): Unstimulated THP-1 cells released very low concentrations of IL-1β, IL-8, and TNF-α. When the cells were stimulated with 10 ng mL^− 1^ LPS a significant increase in the release of all three cytokines was detected. The release of all three cytokines was further increased by co-stimulation with LPS and IFN-γ (10 ng mL^− 1^ each) (mean ± s.d.; *p ≤ 0.05 compared to unstimulated THP-1 cells, ^#^p ≤ 0.05 compared to LPS-stimulated THP-1 cells).

Other groups have demonstrated that increased levels of pro-inflammatory cytokines can negatively affect the barrier integrity of IECs (Watanabe et al., 2004, Satsu et al., 2006). Therefore, we aimed to reduce the background cytokine levels without impairing the differentiation of THP-1 cells by adapting the PMA differentiation protocol. Simplifying the protocol to 24 h PMA-treatment without rest period (hereinafter ‘24 h-differentiated’) significantly decreased the release of IL-1β and IL-8 from unstimulated THP-1 cells by 66% and 87% to 8.3 and 615 pg mL^− 1^, respectively (Fig. 1, black bars), compared to 48 h-differentiated cells (Fig. S2). In addition, TNF-α was below the detection limit (Fig. 1).

However, the shortened differentiation also affected the cytokine response to LPS. After a 4 h exposure to LPS, 24 h-differentiated THP-1 cells released significantly less IL-8 (4125 pg mL^− 1^) and TNF-α (391 pg mL^− 1^) (Fig. 1, white bars) compared to 48 h–differentiated THP-1 cells (11,328 and 1037 pg mL^− 1^, respectively) (Fig. S2). The levels of IL-1β were less affected with 167 pg mL^− 1^ released by 24 h–differentiated THP-1 cells (Fig. 1, white bars) compared to 221 pg mL^− 1^ after 48 h differentiation (Fig. S2).

To compensate for the reduction, 24 h-differentiated THP-1 cells were co-stimulated with LPS and IFN-γ (10 ng mL^− 1^ each). The co-stimulation significantly increased (p ≤ 0.003) the release of all three cytokines compared to stimulation with LPS alone (IL-1β: + 240%, IL-8: + 190%, and TNF-α: + 150%) (Fig. 1, striped bars).

### 3.2 Establishing a stable co-culture using Caco-2 cells and PMA-differentiated THP-1 cells

**Fig. 2:**
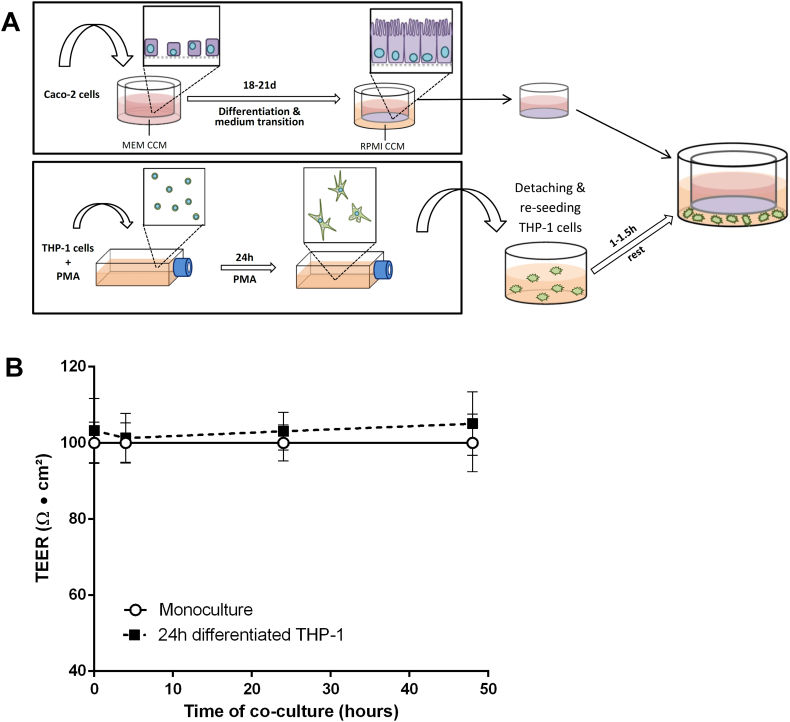
Stable co-culture set-up: (A) Schematic description of the stable co-culture set-up; (B) barrier integrity as TEER of Caco-2 cells over 48 h co-culture with 24 h PMA-differentiated THP-1 cells. The co-culture of Caco-2 cells with 24 h-differentiated THP-1 cells did not induce significant changes in the TEER over a period of 48 h (mean ± s.d.; n = 6).

To develop a co-culture model mimicking the homeostatic intestine it is necessary that the cell lines do not affect each other negatively. For example the THP-1 cells should not be activated to release cytokines or generate NO, nor affect the Caco-2 barrier integrity permanently. A graphic overview of the cell culture and co-culture set-up is given in Fig. 2A.

To initiate the co-culture, the transwell filters with Caco-2 cells were transferred onto well plates containing 24 h-differentiated THP-1 cells without additional manipulation of either cell line. To monitor the effect of THP-1 cells on the Caco-2 barrier the TEER was measured after 4, 24, and 48 h of co-culture. Changes in the Caco-2 environment (e.g. medium change, temperature) resulted in temporary TEER reductions of up to 10% without negatively affecting the overall barrier integrity (data not shown). Under normal conditions, the TEER stabilised after a maximum of 24 h. We, therefore, regarded a TEER reduction of ≤ 10% in the stable co-culture as acceptable, if the barrier integrity was re-established to ≥ 95% after 24 h.

As presented in Fig. 2B, the TEER of Caco-2 barriers co-cultured with 1.8 × 10^5^ 24 h-differentiated THP-1 cells was not significantly different from control monocultures over a period of 48 h. Both the PMA-differentiation protocol and the THP-1 seeding density were crucial for the co-culture stability. Co-cultures established with 48 h-differentiated THP-1 cells (for schematic description see Fig. S4) failed to meet the earlier defined criteria for barrier integrity (Fig. S5), as did co-cultures using increased numbers of 3.99E + 5 24 h-differentiated THP-1 cells (data not shown).

Hereinafter, the term ‘stable co-culture’ refers to a co-culture of Caco-2 and 24 h-PMA-differentiated THP-1 cells.

### 3.3 Development of an inflamed co-culture model mimicking the diseased human intestine

**Fig. 3:**
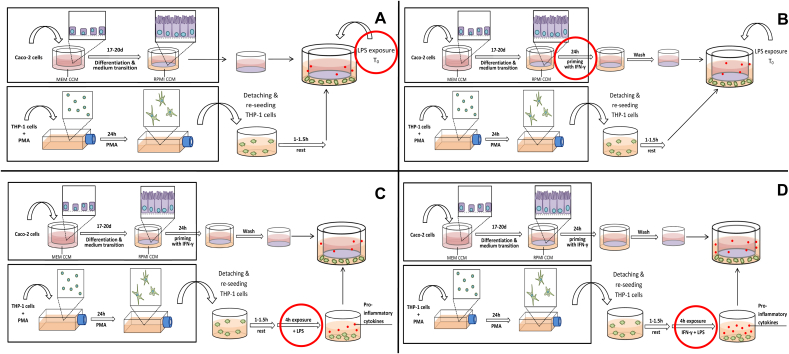
Inflamed co-culture set-up: (A) Step 1, (B) Step 2, (C) Step 3, (D) Step 4 of the inflamed co-culture development: In Step 1 (A), the set-up of the stable co-culture was not altered apart from the addition of LPS to the BL compartment at the start of the co-culture with 24 h PMA-differentiated THP-1 cells. In a second step (B), the Caco-2 barrier was primed with IFN-γ before the start of the co-culture. In Step 3 (C), the THP-1 cells were pre-exposed to LPS 4 h before the initiation of the co-culture and, eventually, pre-treated with LPS and IFN-y to stimulate an uninhibited cytokine response in the cells (Step 4; D). [Changes to the previous set-up are marked by red circles.] (For interpretation of the references to color in this figure legend, the reader is referred to the web version of this article.)

The second aim of this work was to disrupt the stable co-culture through the activation of THP-1 cells and, thereby, induction of an inflammation-like response. To not alter the model unnecessarily, adjustments were introduced in separate steps, which will be described below. A graphic description of the set-up and all changes is given in Fig. 3 (A–D).

#### 3.3.1 Step 1: LPS-stimulation of THP-1 cells

**Fig. 4:**
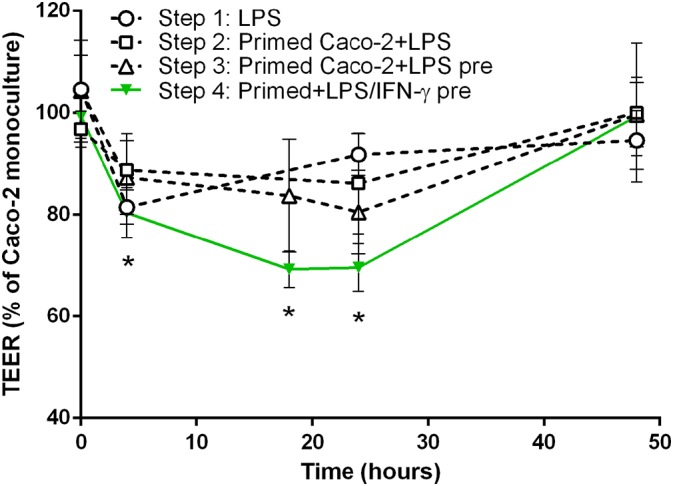
Barrier integrity measured as TEER over 48 h co-culture of Caco-2 cells with differently stimulated THP-1 cells. Step 1: The addition of LPS (10 ng mL^− 1^) to the BL compartment of the stable co-culture set-up at the start of the co-culture did not induce a sufficiently pronounced and prolonged TEER reduction. Step 2: By priming the Caco-2 cell barrier with IFN-γ (10 ng mL^− 1^) before the co-culture and subsequent activation of THP-1 with LPS the barrier disruption could be extended. Step 3: Pre-exposing the THP-1 cells to LPS in advance of the co-culture further increased the TEER reduction. Step 4: Only after the co-stimulation of THP-1 cells with LPS and IFN-γ (10 ng mL^− 1^ each) 4 h prior of the co-culture a sufficiently pronounced, prolonged, and reproducible barrier disruption could be induced (mean ± s.d.; Step 1 t4: n = 2; *p ≤ 0.05 compared to Caco-2 monoculture at corresponding time point; two-sample *t*-test). (For interpretation of the references to color in this figure, the reader is referred to the web version of this article.)

At first, the stable co-culture set-up was not altered except for the addition of 10 ng mL^− 1^ LPS to the BL compartment at the start of the co-culture (t_0_) (Fig. 3A). Subsequent to an early 19% reduction in TEER after 4 h (Fig. 4, dotted line circles) the barrier integrity quickly re-established to > 90% of the Caco-2 monoculture after 24 h.

#### 3.3.2 Step 2: IFN-γ-priming of Caco-2 cells and LPS stimulation of THP-1 cells

Since the LPS stimulation alone was not sufficient to adequately disrupt the barrier, the priming effect of IFN-y described by Wang et al. (2005) was exploited. Wang et al. (2005) demonstrated that the Caco-2 barrier integrity was impaired when both IFN-γ and TNF-α were present simultaneously or the Caco-2 cells were first exposed to IFN-γ and subsequently to TNF-α. Based on these findings, Caco-2 barriers were cultured with 10 ng mL^− 1^ IFN-γ in the BL compartment 24 h in advance of the co-culture (Fig. 3B). The co-culture was initiated as before and the THP-1 cells were challenged with the addition of 10 ng mL^− 1^ LPS to the BL compartment. As shown in Fig. 4 (dotted line, squares), the IFN-γ-priming prolonged the barrier disruption induced by activated THP-1 cells. With a maximum TEER reduction of 14% the effect was, however, not sufficient to meet the defined requirements.

#### 3.3.3 Step 3: IFN-γ-priming of Caco-2 cells and LPS pre-stimulation of THP-1 cells

Based on the results obtained from LPS-stimulated THP-1 monocultures the release of TNF-α should have been sufficient to induce a significant reduction in TEER as described earlier (Van De Walle et al., 2010, Wang et al., 2005). However, a significantly lower induction of cytokine release was noted in LPS-stimulated co-cultures compared to THP-1 monocultures (Fig. S6). Additionally conducted experiments suggested that a down-regulation of the macrophage-driven stress response occurs in presence of Caco-2 cells (Fig. S7). To overcome the impact of the Caco-2 cells, the THP-1 cells were pre-stimulated with LPS for 4 h before the start of the co-culture (Fig. 3C). The pre-exposure to LPS increased and pro-longed the barrier disruption to 20% of the monoculture after 24 h of co-culture (Fig. 4, dotted line, triangles). This set-up fulfilled the minimum requirements, but remained close to the lower limits of the criteria. Furthermore, as indicated by the high standard deviations, the reproducibility of the results was poor.

#### 3.3.4 Step 4: IFN-γ-priming of Caco-2 cells and LPS/IFN-γ pre-stimulation of THP-1 cells before the co-culture

To increase the barrier disrupting effect and its reproducibility, IFN-γ was introduced to co-stimulate the THP-1 cells. In earlier experiments the co-stimulation with IFN-γ and LPS was shown to enhance the cells' cytokine response (Fig. 1). The cells were pre-stimulated with LPS and IFN-γ (10 ng mL^− 1^ each) simultaneously 4 h before the co-culture with IFN-γ-primed Caco-2 cell layers was initiated (Fig. 3D). The co-stimulation induced a sufficiently pronounced barrier disruption to meet the defined criteria (Fig. 4, green line). Within 4 h of co-culture, the TEER decreased significantly to 80 ± 4.8% of the Caco-2 monoculture control and further to 69 ± 3.5% and 69 ± 4.7% after 18 h and 24 h, respectively. Interestingly, the TEER had re-established to ~ 99 ± 7.2% of the control after 48 h of co-culture without additional manipulation of the system.

Hereinafter, the term ‘inflamed co-culture’ refers to a set-up according to Step 4: 24 h-differentiated THP-1 cells, 4 h pre-exposed to LPS and IFN-γ, in co-culture with 24 h IFN-γ-primed Caco-2 cells.

### 3.4 Characterisation and comparison of the co-culture models

The two co-culture models of the healthy and inflamed intestine were compared in terms of functional and morphological characteristics, cellular damage, as well as the release of cytokines and formation of NO.

As presented above, the stable co-culture of Caco-2 cells with 24 h-differentiated THP-1 cells did not induce a reduction in TEER over a period of 48 h, whereas in presence of LPS/IFN-γ-activated THP-1 cells, a significant temporary barrier disruption was induced. We assessed whether this reduction in TEER caused an enhanced paracellular passage of the dye LY. With LY dissolved in MEM, the inflamed model co-culture generated similar TEER results as the corresponding control (Fig. S8). No increase in LY translocation was measured compared to the Caco-2 monoculture and stable co-culture controls (Fig. S9). All culture models were virtually impermeable to the dye.

**Fig. 5:**
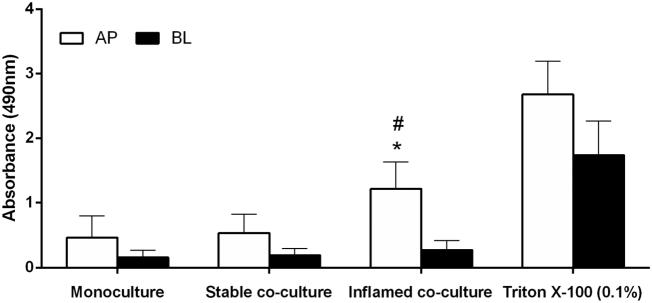
LDH release after 48 h Caco-2 monoculture, stable, and inflamed co-culture: No significant increase in LDH release was detected in the stable co-culture compared to the Caco-2 monoculture. In the inflamed model, the LDH activity was significantly enhanced in the AP compartment. The AP activity of LDH in the inflamed co-culture was equal to 41% of the positive control using Triton X-100 (mean ± s.d.; Triton X-100 (0.1%): n = 2 of 2 performed, *p ≤ 0.05 compared to Caco-2 monoculture; ^#^p ≤ 0.05 compared to stable co-culture).

The quantification of LDH release was included to identify potential cytotoxic effects of THP-1 cells in the stable co-culture, as well as the impact of THP-1 activation in the inflamed model. The LDH release in Caco-2 monocultures served as a baseline: very low enzymatic activity could be detected in both apical (AP) and basolateral (BL) samples. In the stable co-culture, no significant increase in LDH release was detected in either compartment compared to the monoculture control (Fig. 5).

In the inflamed co-culture, the LDH activity was strongly increased (260%) in the AP compartment and moderately increased (150%) in the BL compartment (Fig. 5), indicating the occurrence of necrotic cell death. In AP supernatants, the increase was statistically significant compared to both the monoculture and stable co-culture (p ≤ 0.001).

**Fig. 6:**
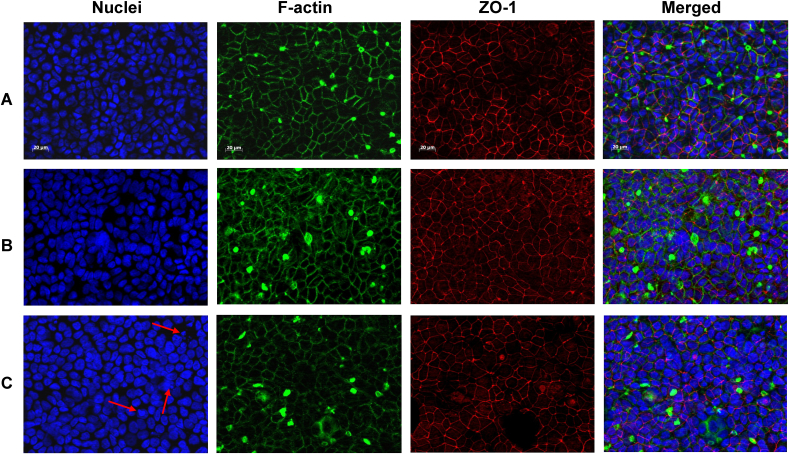
Immunocytochemical staining of nuclei, F-actin, and ZO-1 after 48 h (A) Caco-2 monoculture, (B) stable co-culture or (C) inflamed model co-culture. (For interpretation of the references to color in this figure, the reader is referred to the web version of this article.)

Since the barrier integrity measured by TEER was fully re-established after 48 h of co-culture we assumed that no excessive cell death occurred. The microscopic analysis confirmed that the co-culture with 24 h-differentiated THP-1 cells did not affect the Caco-2 barrier (Fig. 6). No variation between the stable co-culture barrier (Fig. 6B) and the Caco-2 monoculture (Fig. 6A) was noted. In the inflamed co-culture barrier (Fig. 6C), no gaps or reduced number of nuclei were observed. However, the TJ network (red) and cytoskeleton (green) seemed irregular and less organised compared the monoculture and stable co-culture. Furthermore, an occurrence of nuclear fragmentation was observed and the nuclear surface area appeared increased compared to the monoculture and stable co-culture (Fig. 6C, red arrows). Additional imaging of DAPI-stained Caco-2 barriers using the IN Cell Analyzer confirmed our initial observation (Fig. S10, red arrows), showing a higher occurrence of condensed and fragmented nuclei.

**Fig. 7:**
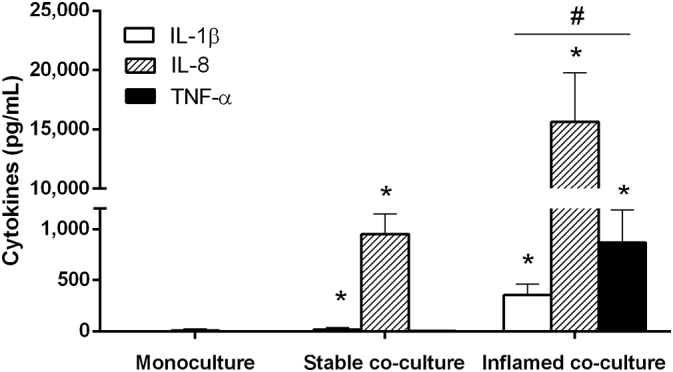
Release of pro-inflammatory cytokines after 4 h Caco-2 monoculture, stable or inflamed model co-culture: Whereas IL-1β, IL-8 and TNF-α were nearly absent in Caco-2 monocultures, a significant amount of IL-8 was detected in stable co-cultures. IL-1β was slightly increased, however, TNF-α remained undetectable. In the inflamed model co-culture, all three cytokines were significantly increased compared to the monoculture and stable co-culture (mean ± s.d.; *p ≤ 0.05 compared to Caco-2 monoculture; ^#^p ≤ 0.05 compared to stable co-culture).

Finally, we compared the cytokine release and NO generation in the Caco-2 monoculture and both co-culture models. The cytokine release was analysed using supernatant samples from the BL compartment after 4 h or 28 h of monoculture, stable, or inflamed co-culture. TGF-β1 was expressed at very low concentrations (< 10 pg mL^− 1^; data not shown) and no differences in its expression were observed between the culture models. The results for IL-1β, IL-8, and TNF-α are summarised in Fig. 7.

In Caco-2 monocultures, IL-8 was expressed at low concentrations of 61 ± 64 pg mL^− 1^. The release of IL-1β and TNF-α were below the detection limit. In the stable co-culture, IL-1β was slightly increased to 29 ± 18 pg mL^− 1^, but TNF-α remained below the detection limit. Even though the change in PMA differentiation protocol reduced the IL-8 expression of THP-1 cells significantly, a clear release of ~ 1000 pg mL^− 1^ was detected after 4 h of stable co-culture.

Compared to both the Caco-2 monoculture and stable co-culture a clear release of all three cytokines was detected in the inflamed model. In response to LPS/IFN-γ co-stimulation, the levels of IL-1β and TNF-α increased significantly (p ≤ 0.001) to 350 and 870 pg mL^− 1^, respectively (Fig. 7). Most striking was the increase in IL-8 to > 15,000 pg mL^− 1^ (Fig. 7).

Similar results were obtained for the quantification of TNF-α, IFN-γ, IL-6, MIP-1α, and MCP-1 after 28 h. All cytokines were close to or below the detection limit in the Caco-2 monoculture (Fig. S11A, B). In the stable co-culture, the releases of MCP-1 and MIP-1α (Fig. S11A, B) were elevated, but only significantly for MIP-1α (Fig. S11B). All cytokines were significantly enhanced in the inflamed co-culture compared to the Caco-2 monoculture and stable co-culture (Fig. S11A, B). Both IL-4 and GM-CSF were released at negligible quantities in all three culture models (Fig. S11A).

The generation of NO was indirectly quantified *via* the detection of NO_2_^−^. The background levels of NO_2_^−^ in Caco-2 monocultures were low or undetectable (Fig. S12). Since the detection limit of the Griess Reaction is around 3 μM of NO_2_^−^ (Malinski et al., 1996) results below this concentration were regarded as negligible. After 48 h of stable co-culture, no change in NO_2_^−^ was detected compared to the monoculture. In the supernatants of the inflamed co-culture model, an increase to 5.5 ± 1.3 μM and 5.3 ± 1.4 μM NO_2_^−^ was quantified in the AP and BL compartment, respectively, which was significant (p ≤ 0.001) compared to both the Caco-2 monoculture and stable co-culture (Fig. S12).

### 3.5 Activation of the stable co-culture

To test the stable co-culture's ability to react to pro-inflammatory stimuli, an increase in cytokine release after apical exposure to a stressor had to be documented. The stable co-culture was exposed for 4 h to 2.5 mM EDTA on the AP and BL side to reduce Caco-2 cell-to-cell attachment (Meng and Takeichi, 2009) and enable apically added LPS to translocate to the BL compartment to stimulate the THP-1 cells.

After the addition of EDTA at t_0_, the TEER rapidly decreased by ~ 80% over 4 h (Fig. S13). Apical exposure to LPS alone (10 ng mL^− 1^) or LPS and IFN-γ (10 ng mL^− 1^ each) did not induce a comparable TEER reduction in the Caco-2 monoculture and stable co-culture (Fig. S14A, B). The AP supernatant was exchanged after 4 h without noticeable effect on the TEER, which suggested an extensive and permanent damage of the cell barrier.

**Fig. 8:**
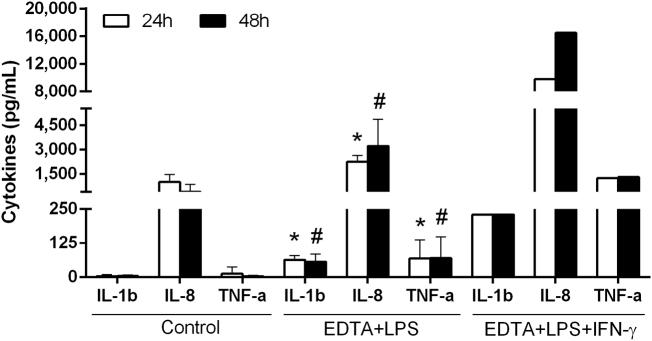
Cytokine release after 24 and 48 h stable co-culture without (Control) and with AP exposure to EDTA (2.5 mM) and LPS (10 ng mL^− 1^) or EDTA, LPS, and IFN-γ (10 ng mL^− 1^): After 24 h exposure, a clear increase in IL-8 occurred in the EDTA + LPS-exposed condition compared to the control. Also IL-1β and TNF-α were elevated, but remained at comparably low levels. In stable co-cultures exposed to EDTA, LPS, and IFN-γ the release of all three cytokines was markedly increased after 24 and 48 h of exposure, reaching similar levels as measured in the inflamed model co-culture. (mean ± s.d.; EDTA + LPS + IFN-γ: n = 1 of 1 performed with three technical replicates; *p ≤ 0.05 compared to corresponding 24 h control; ^#^p ≤ 0.05 compared to corresponding 48 h control; unpaired two-sample *t*-test).

The release of IL-8 was significantly elevated in the EDTA/LPS-exposed stable co-culture compared to the stable co-culture control after 24 (p ≤ 0.001) and 48 h (p = 0.01) of exposure (Fig. 8). The release of IL-1β and TNF-α increased marginally but significantly (IL-1β: p ≤ 0.001; TNF-α: p ≤ 0.037) in response to EDTA and LPS. When IFN-γ (10 ng mL^− 1^) was included in the exposure the levels of all three cytokines were markedly increased and reached concentrations similar to those recovered from the inflamed co-culture. The exposure of Caco-2 monocultures to EDTA and LPS, as well as exposure of THP-1 monocultures to EDTA did not result in elevated concentrations of IL-8 (Fig. S15A, B).

## 4 Discussion

An *in vitro* co-culture model of intestinal epithelial cells and macrophages that can mimic the intestine in a healthy or a controlled inflamed state was developed.

To mimic the conditions of the healthy intestine, a co-culture of Caco-2 and THP-1 was established in which the cell lines coexisted without adverse interference. No significant changes in TEER, LDH release, cytokine release, and NO generation were observed in stable co-cultures of Caco-2 and 24 h-differentiated THP-1 cells.

Over the last 15 years, several *in vitro* co-culture models have been developed to mimic the intestine with representation of the immune system. For the development of these immuno-active co-cultures both primary mononuclear cells (Bisping et al., 2001, Leonard et al., 2010) and cell lines from rodent (Tanoue et al., 2008) or human origin (Watanabe et al., 2004, Susewind et al., 2016, Moyes et al., 2010) have been used. To represent the intestinal barrier, most of the models made use of the Caco-2 cell line. Compared to other intestinal epithelial cell lines, the Caco-2 cell line is advantageous due to its spontaneous differentiation to an enterocyte-like phenotype, formation of TJ network, and good functional correlation to human intestinal tissue (Rubas et al., 1993, Rubas et al., 1996). In contrast to primary IECs the cell line is easily accessible and maintainable (Grajek and Olejnik, 2004), which made the Caco-2 cell line our first choice. The use of THP-1 cells was a compromise. The great majority of macrophages found in the homeostatic intestine do not express several response receptors, including the LPS-receptor CD14, and are characterised by a striking inflammatory *anergy*, which describes the lack of pro-inflammatory reactions towards non-self-antigens (Smythies et al., 2005). To be able to study the pro-inflammatory potential of substances, as well as to establish the model of the inflamed intestine the macrophages had to be able to respond to stressors, here LPS.

Our results are in contrast to the Caco-2/THP-1 co-cultures of other groups. Both Watanabe et al. (2004) and Satsu et al. (2006) described the induction of barrier-disruptive effects by unstimulated THP-1 cells, indicated by a permanent strong reduction in TEER (30% after 24 h, 80% after 48 h) and LDH release. The disruptive effect was attributed to the spontaneous release of TNF-α by THP-1 cells. Also Kanzato et al. (2001) and Moyes et al. (2010) reported a significant and permanent TEER reduction of Caco-2 barriers in presence of unstimulated THP-1 cells. However, they did not observe (Kanzato et al., 2001) or investigate (Moyes et al., 2010) cytotoxicity.

These different outcomes might be explained with several variations in the cell treatment and co-culture establishment. Compared to our study, Satsu and colleagues differentiated THP-1 cells with higher PMA concentrations (200 *vs.* 100 nM here) and for an extended period of time (4 days *vs.* 24 h here). The differentiation protocol strongly affects the cell characteristics and response to stressors (Daigneault et al., 2010), especially THP-1-differentiation with PMA which has been demonstrated to result in highly variable outcomes between laboratories (Xia et al., 2013). The results of Satsu et al. showed a significantly increased release of pro-inflammatory cytokines after PMA-treatment without additional stimulation of THP-1 cells. As the group detected both TNF-α and IFN-γ, it is possible that the Caco-2 barrier was disrupted through the same mechanism exploited in our study, yet in an uncontrolled manner. Also in our study, an increased release of pro-inflammatory cytokines by unstimulated THP-1 cells was measured after the 48 h PMA-differentiation, which resulted in an instable Caco-2 barrier. However, this mainly concerned IL-8 and only to a lesser extent TNF-α.

Another factor that might have influenced the outcomes is the seeding density of THP-1 cells. In the study by Satsu et al. (2006), THP-1 cells were seeded at 1.6-times higher numbers than we used. Similar outcomes were reported by Moyes and colleagues. The group treated the THP-1 cells the same way as we did but established the co-culture with a higher THP-1 cell density of 1.05E + 5 cells cm^− 2^ compared to 4.7E + 4 cells cm^− 2^ well surface used here (Moyes et al., 2010). During the development of our co-culture model, we observed that a 2.2-fold increase of THP-1 cells lead to more instable TEER results and increased concentrations of pro-inflammatory cytokines (data not shown). Interestingly, neither of these points can explain the TEER reduction reported by Kanzato et al. (2001). Compared to this work, Kanzato and colleagues used a lower macrophage density (3.9E + 4 cells cm^− 2^) and refrained from differentiating the THP-1 cells. In combination with the findings of other groups our observations highlight the variability of THP-1 cells in the response to PMA, as well as how the cells' seeding density can impact the outcomes of a co-culture with IECs. These variabilities might lead to difficulties in the establishment of the co-culture model among laboratories.

The influence of the THP-1 cell number on the co-culture stability could be related to the immuno-stabilising effects of the Caco-2 cells. Apart from their barrier functions, IECs are well-known to play an active role in the regulation of homeostasis in healthy intestinal tissue. They discriminate between commensal “self” and harmful “non-self” antigens in the intestinal lumen (Rakoff-Nahoum et al., 2004) and control the responsiveness of immune cells in the lamina propria (Rimoldi et al., 2005, Nathens et al., 1995). Without the IEC-mediated down-regulation of the immune system, the intestine would likely be subject to constant inflammatory processes caused by non-harmful antigens. The ability to down-regulate the responsiveness of immune cells has been demonstrated *in vitro* using Caco-2 cells (Parlesak et al., 2004). It is possible that the cells are only able to control the system up to a certain threshold number of macrophages. When this threshold is exceeded, the down-regulating mechanisms of Caco-2 cells might not be sufficient anymore and the system loses its equilibrium. These results underline the importance of establishing adequate ratios between cell types when developing a co-culture model.

Whereas the established ratio between IECs and macrophages was of utmost importance for the successful development of the stable co-culture, it complicated the development of the inflamed model. Inflamed intestinal tissue is characterised by reduced barrier integrity, infiltration of pro-inflammatory leukocytes, and high local concentrations of pro-inflammatory cytokines, reactive oxygen species and reactive nitrogen species (Turner, 2009, Pavlick et al., 2002). In addition to CD14^−^ intestinal macrophages, large numbers of neutrophils and CD14^+^ macrophages infiltrate the intestine at sites of inflammation. Therefore, it was necessary to introduce several adjustments to the set-up of the stable condition to obtain a co-culture displaying numerous hallmarks of intestinal inflammation. The inflamed co-culture was characterised by a significant but temporary barrier disruption, occurrence of necrotic and apoptotic cell death, as well as high levels of pro-inflammatory mediators, including cytokines (TNF-α, IL-1β, IL-8, MCP-1, MIP-1α, IFN-γ, and IL-6) and NO.

Few other groups documented *in vitro* models of the inflamed intestine. However, most displayed uncontrolled, spontaneous inflammation-like processes (Watanabe et al., 2004, Satsu et al., 2006, Moyes et al., 2010) or used non-physiological stressors for the induction (Detzel et al., 2015). Only one other group created a model using Caco-2 cells in combination with primary macrophages and dendritic cells (DCs) (Leonard et al., 2010) or cell line-derived macrophages and DCs (Susewind et al., 2016) that can be used both in homeostatic and controlled inflamed states. In contrast to our approach, both Leonard et al. and Susewind et al. chose to embed the leukocytes in a collagen layer under the Caco-2 barrier. This set-up might enable more realistic interactions between the cells types by allowing the immune cells to move in 3 dimensions and interact with the IECs. However, embedding the immune cells in collagen might affect their biological activity (Vaday and Lider, 2000) as well as the detection of released pro-inflammatory mediators. Our intention was to use the model to study the uptake and potential toxicity of nanomaterials. Therefore, we considered the use of collagen less suitable. Furthermore, both Leonard et al. and Susewind et al. decided to stimulate the co-culture using IL-1β. The exposure to IL-1β caused a clear reduction in TEER, but the increase in IL-8 was significantly lower compared to the results presented here. Furthermore, the group did not observe cytotoxic effects measured by LDH release, and the re-establishment of barrier integrity required a medium exchange (Susewind et al., 2016).

We focused on *E. coli*-derived LPS as alternative stimulus and chose to directly expose the THP-1 cells to provoke an inflammation-like response. LPS is a heat-stable toxin associated with the outer membranes of gram-negative bacteria and belongs to the most studied pathogen-associated molecular patterns. It is ubiquitously present in the intestinal lumen and known for its involvement in intestinal inflammation (Guo et al., 2013). Caco-2 cells express the LPS binding receptor CD14 as well as Toll-like Receptor (TLR)-2 (Good et al., 2012). But in contrast to THP-1 cells they lack the expression of TLR4 which is essential for LPS-induced signalling (Tamai et al., 2003, Melmed et al., 2003, Bocker et al., 2003). Whereas the responsiveness of IECs is not influenced by the expression of CD14 or TLR2, it was shown to depend on the presence of TLR4 (Bocker et al., 2003). Therefore, BL exposure of the co-culture mimics the pathophysiological scenario of bacterial antigens translocating across the IEC barrier and subsequently activating cells of the GALT (Turner, 2009) while stimulation of Caco-2 cells with low concentrations of LPS on the AP side should not result in any significant effect. Indeed, in our model the AP exposure to LPS did not induce barrier disruption or cytokine release in Caco-2 monocultures or stable co-cultures suggesting that no translocation of LPS across the IEC barrier occurred. In contrast, a co-culture model of Caco-2 and peripheral blood mononuclear cells (PBMCs) resulted in significantly increased production of pro-inflammatory cytokines after the AP exposure to a non-pathogenic *E. coli* strain, albeit to a lesser extent than the direct exposure of PBMCs (Parlesak et al., 2004). However, *E. coli* bacteria can interact with IECs and translocate across the epithelium to reach the BL compartment, whereas *E. coli*-derived LPS remains on the luminal side of the IEC barrier (Neal et al., 2006).

However, mere addition of LPS to the BL compartment was not sufficient to induce the desired barrier disruption and cytokine response. Though 10 ng mL^− 1^ LPS were able to activate THP-1 monocultures it failed to do so in Caco-2/THP-1 co-cultures. Presumably, the Caco-2 cells down-regulated the pro-inflammatory response of THP-1 cells, as occurs *in vivo* and was observed by others *in vitro* (Parlesak et al., 2004). The control exerted by the Caco-2 cells could be bypassed by pre-exposing THP-1 cells to LPS in advance of the co-culture and integrating IFN-γ in the system through Caco-2 barrier-priming with IFN-γ and IFN-γ-stimulation of THP-1 cells. Wang et al. (2005) showed that ‘priming’ of Caco-2 cells with IFN-γ induces an up-regulation of both TNF-α receptor (TNFR) 1 and 2, which are located at the BL cell membrane (Li et al., 1998). Binding of TNF-α to TNFR2 but not TNFR1 leads to an increased expression of myosin light chain kinase (MLCK) (Blumberg, 2009), which in turn results in amplified phosphorylation of myosin light chain (MLC) (Zhou et al., 2005). Phosphorylation of MLC, leading to contraction of the actomyosin ring, has been demonstrated to regulate intestinal epithelial permeability by re-organisation of TJ proteins. This re-organisation of TJ protein causes increased paracellular permeability and a strong reduction in TEER (Turner et al., 1997, Wang et al., 2005, Zhou et al., 2005). Up-regulated MLCK was shown to be prevalent in biopsies taken from patients with active IBD (Blair et al., 2006) and to be involved in the pathogenesis of experimental colitis (Weber et al., 2010).

To further prove the stable co-culture's ability to react to pro-inflammatory stimuli, we tested whether apical exposure to a stressor can induce cytokine release. In this case, we decided to adapt the approach described by Detzel et al. (2015). Briefly, the group exposed C2BBe1 cell layers to 0.01% dimethyl palmitoyl ammonio propanesulfonate (PPS) to reduce TJ integrity and allow for the translocation of apically added LPS. Instead of PPS, we exposed the stable co-culture to EDTA on the AP and BL side to reduce Caco-2 cell-to-cell attachment (Meng and Takeichi, 2009) and enable apically added LPS to translocate to the BL compartment to stimulate the THP-1 cells. The exposure to EDTA caused a permanent damage to the epithelial barrier. Apical addition of LPS in these conditions induced the release of IL-8, TNF-α, and IL-1β. However, it became clear that the inclusion of IFN-γ was crucial to achieve similar levels of pro-inflammatory cytokines as measured in the inflamed model co-culture.

The inflamed co-culture featured a significantly increased release of LDH, and the occurrence of either fragmented or swollen nuclei. The nuclear integrity and mean nuclear size are important indicators for cellular health and integrity. The condensation of chromatin and nuclear fragmentation are late events in apoptotic cell death (Tounekti et al., 1995, Collins et al., 1997), whereas cell and nuclear swelling, as well as LDH release are associated with necrotic processes (Labbé and Saleh, 2011). Apoptotic processes could have originated from the IFN-γ-priming of Caco-2 cells before the start of the co-culture. The extended exposure of Caco-2 cells to IFN-γ has been shown to induce apoptosis and to decrease cell proliferation (Nava et al., 2010). On the other hand, the cell damage might be directly linked to the co-culture with stimulated THP-1 cells. TNF-α is known for its ability to induce apoptotic mechanisms (Miura et al., 1995), whereas NO can be involved in both apoptotic and necrotic cell death (Liu et al., 2003, Bonfoco et al., 1995).

The induction of apoptosis does not explain the elevated LDH activity measured earlier (Chan et al., 2013), but necrosis does not feature the condensation of chromatin (Vanden Berghe et al., 2010) as was observed here. Instead, the results might indicate apoptosis-induced secondary necrosis, which can occur when the scavenging capacities of phagocytising cells are overwhelmed or impaired (Silva et al., 2008). In contrast to primary necrosis, secondary necrosis features nuclear shrinkage and severe fragmentation of the nucleus, as well as cytoplasmic swelling (Silva, 2010). As mentioned above, pro-inflammatory cytokines and NO can induce apoptosis in IECs, whereas the LDH release could have been caused by TNF-α-mediated necrotic processes. The induction of necrotic processes accompanied by cellular swelling could explain the re-establishment of barrier integrity in the inflamed co-culture, which was initially interpreted as restoration of the TJ network. Instead the increase in TEER might have been caused by a reduction in intracellular space caused by cellular swelling as part of necrotic cell death.

In conclusion, a human cell line-based co-culture model of the human intestine was established which can mimic the intestine in homeostatic or controlled inflamed states. The stable co-culture can be applied to study the pro-inflammatory potential of substances provided the critical role of IFN-γ in the induction of inflammation is considered. The priming of Caco-2 cells together with the stimulation of THP-1 cells by LPS and IFN-γ provoked an inflammation-like response which is highly comparable to major pathophysiological mechanisms of intestinal inflammation. With a disrupted IEC barrier, the presence of high concentrations of pro-inflammatory cytokines, the generation of NO, and occurrence of cell death several hallmarks of intestinal inflammation are displayed. Having the realistic cell ratios together with the controlled induction of inflammation using a relevant pathophysiological mechanism, this *in vitro* model offers unique side-by-side experimental possibilities to study the effect of compounds in relation to the health status, as well as pro- or anti-inflammatory properties of substances.

## Competing interests

No competing interests declared.

## Author contributions

A.K., A.K.-O. and V.S. conceived and designed the experiments. A.K., P.U., S.G. and N.K. supported the organisation of the project and performed the experiments. A.K., P.U. and N.K. analysed the data. A.K. and A.K.-O. wrote the paper. All contributing authors have read and approved the final version of the manuscript.

## Funding

Angela Kämpfer's work was supported by a PhD grant from the European Commissions' Joint Research Centre (contract number: 2012-IPR-I-20-00646).

## Transparency document

Transparency document Image 1

## Appendix A Supplementary data

Supplementary material Image 2
