# Supplementary material for: Development of an in vitro co-culture model to mimic the human intestine in healthy and diseased state
Source: Toxicol In Vitro. 2017 Dec;45:31–43. doi: 10.1016/j.tiv.2017.08.011 (PMC5744654; doi:10.1016/j.tiv.2017.08.011)
Supplement: Supplementary file 2 — Supplementary material [file mmc2.docx]

**Supplementary Information**

**Development of an *in vitro* co-culture model to mimic the human intestine in healthy and diseased state**

Angela A.M. Kämpfer^1,2^, Patricia Urbán^1^, Sabrina Gioria^1^, Nilesh Kanase^2^, Vicki Stone^2^, Agnieszka Kinsner-Ovaskainen^1^

^1^European Commission Joint Research Centre, Directorate F - Health, Consumers and Reference Materials, Via E. Fermi 2749, 21027, Ispra (VA), Italy

^2^Nano-Safety Research Group, School of Engineering and Physical Sciences, Heriot-Watt University, Edinburgh EH14 4AS, United Kingdom

*Corresponding Author:

Agnieszka Kinsner-Ovaskainen

European Commission Joint Research Centre, Directorate F - Health, Consumers and Reference Materials, Via E. Fermi 2749, TP 127, 21027, Ispra (VA), Italy

Email: [agnieszka.kinsner-ovaskainen@ec.europa.eu](mailto:agnieszka.kinsner-ovaskainen@ec.europa.eu)

**Supplementary Information**

Unless stated otherwise, the results represent the mean of three independent experiments. One-way ANOVA with post-hoc Dunnett’s test was used for the statistical analysis, unless explicitly stated otherwise. A p-value of ≤0.05 was accepted as statistically significant. No symbolic differences was made between p=0.05-0.001.

**Table S1. Medium composition in the BL compartment over 21 days of Caco-2 culture**

| **Days of culture** | **Caco-2 medium (%)** | **THP-1 medium (%)** |
| --- | --- | --- |
| 0 | 100 | 0 |
| 2 | 100 | 0 |
| 5 | 66.6 | 33.6 |
| 7 | 66.6 | 33.3 |
| 9 | 50 | 50 |
| 12 | 50 | 50 |
| 14 | 33.3 | 66.6 |
| 16 | 33.3 | 66.6 |
| 19 | 0 | 100 |
| 21 | 0 | 100 |





**Fig. S1. TEER development of Caco-2 cells over 21 days of culture:** The Caco-2 cells reached the maximum TEER between 12-15 days post-seeding with 510 ± 45 Ω•cm². Subsequently, the barrier resistance decreased again to 396±28 Ω•cm² at day 21. (mean ± s.d., Day 2-9: n=2; Day 12-21: n=3)





**Fig. S2. Cytokine release of 48h PMA differentiated THP-1 cells without stimulation or after 4h exposure to LPS:** After 48h differentiation with PMA, THP-1 cells readily released high concentrations of IL-8 (>4,400 pg mL^-1^). The release of TNF-α was overall less but still detectable in most samples. After 4h stimulation with 10 ng/mL LPS (white bars) the release of all three cytokines was markedly increased but most pronounced for IL-8 (>11,000 pg mL^-1^) and TNF-α (~1,000 pg mL^-1^). (mean ± s.d. n=4; *p≤0.05 compared to unstimulated 48h differentiated THP-1 cells; unpaired two-sample t-test)





**Fig. S3. Cytokine release of untreated THP-1 cells without stimulation or after 4h stimulation with LPS:** Untreated THP-1 cells released very low concentrations of pro-inflammatory cytokines (black bars). After 4h of stimulation with 10 ng mL^-1^ LPS the release of IL-8 and TNF-α increased significantly but were clearly lower compared to the cytokine release of 48h PMA-differentiated THP-1 cells. The concentrations of IL-1β remained low even after exposure to LPS. (mean ± s.d.; IL-8: n=1 with three technical replicates of 1 experiment performed; two-sample t-test)


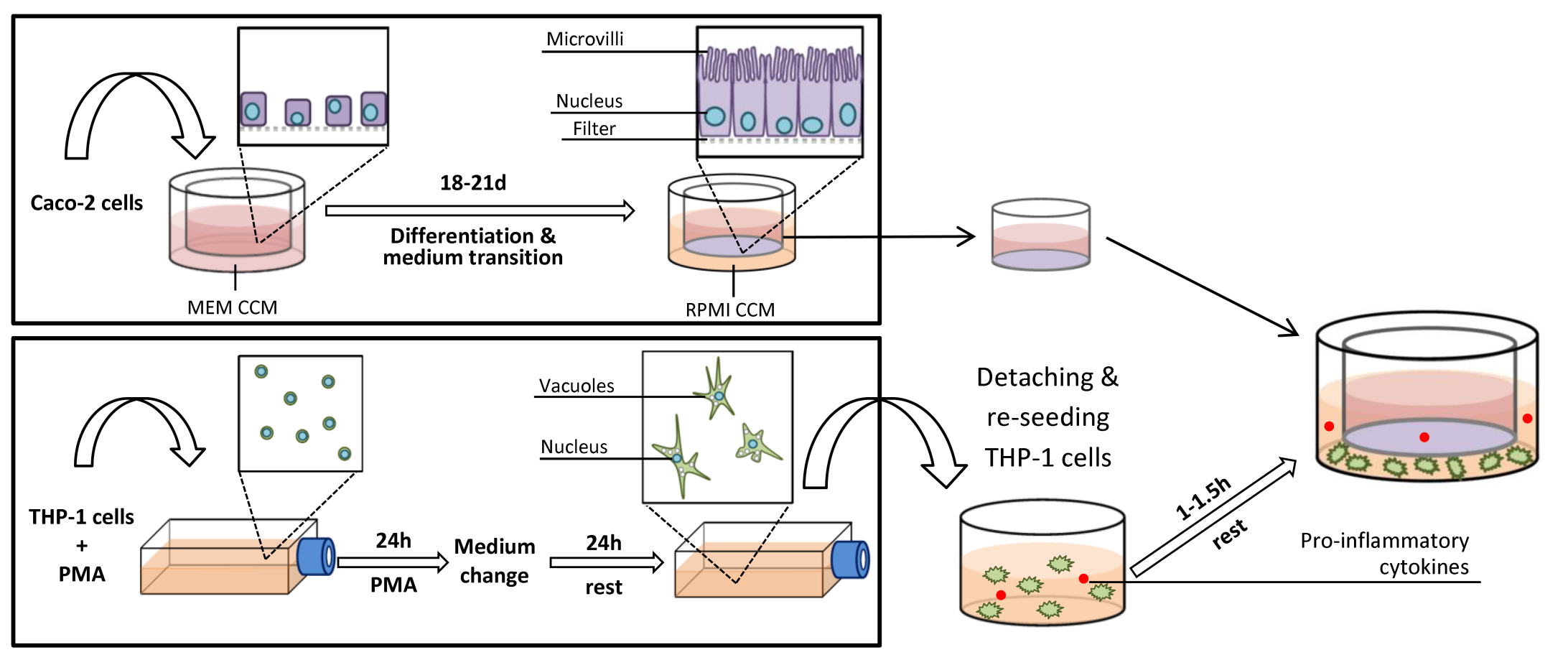


**Fig. S4. Schematic overview: stable co-culture set-up with 48h differentiated THP-1 cells.**


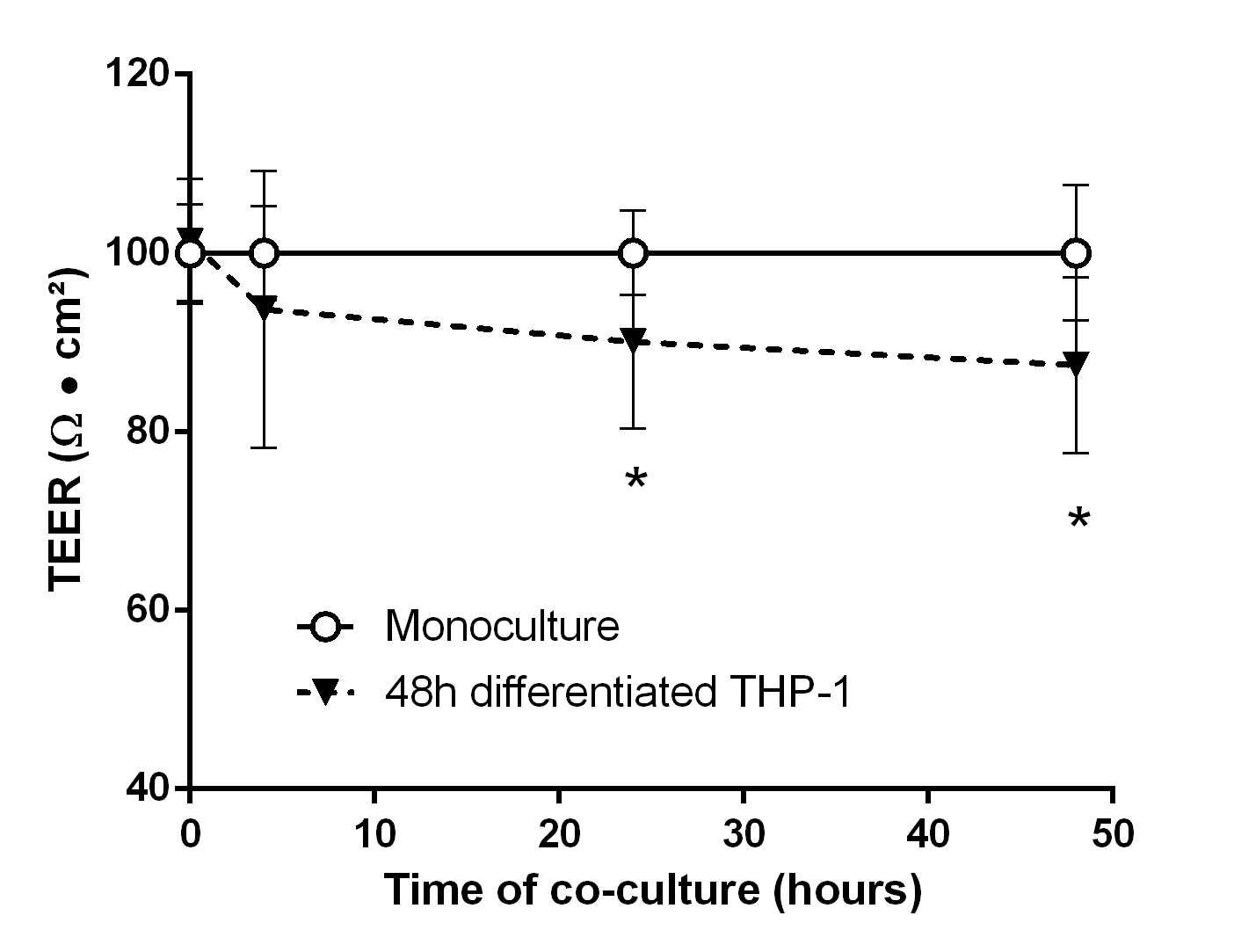


**Fig. S5. Barrier integrity measured as TEER over 48h co-culture with 48h PMA-differentiated THP-1 cells** (mean ± s.d. of n=6; *p≤0.05 compared to Caco-2 monoculture): No significant reduction in TEER was observed at 4h after the start of the co-culture. The barrier integrity was significantly decreased after 24 and 48h to 90±9 and 87±9 Ω•cm², respectively.





**Fig. S6. Cytokine release after 4h LPS stimulation in 24h-differentiated THP-1 monocultures and co-culture with Caco-2 cells:** THP-1 monocultures stimulated with 10 ng mL^-1^ LPS for 4h (white bars) released noticeable concentrations of IL-1β, IL-8, and TNF-α (307±118, 6,643±2,185, and 658±307 pg mL^-1^, respectively). When THP-1 cells were brought in co-culture with Caco-2 cells and subsequently exposed to 10 ng mL^-1^ LPS for 4h (black bars), the release of all three cytokines was significantly lower (22±11, 5,164±1,093, and 71±40 pg mL^-1^ for IL-1β, IL-8, and TNF-α, respectively. (mean ± s.d.; Monoculture n=4, Co-culture: n=2; *p≤0.05 compared to LPS stimulated monoculture, two-sample t-test)

| **A** |  |
| --- | --- |
| **B** |  |

**Fig. S7. Cytokine release (A) after 4h LPS (10 ng mL^-1^) pre-exposure in 24h-differentiated THP-1 monocultures, and (B) after 4h co-culture with unstimulated (PMA control), LPS stimulated (10 ng mL^-1^, LPS exposed at T_0_) or LPS pre-exposed (10 ng mL^-1^) THP-1 cells:** The exposure to LPS induced a string release of IL-1β, IL-8, and TNF-α in THP-1 monocultures (A). The LPS-pre-exposed THP-1 cells were brought in co-culture with Caco-2 cells (B) and the cytokine levels were measured again after 4h (LPS pre-exposed). The concentrations of IL-1β and TNF-α remained constant, whereas the release of IL-8 nearly doubled (7,779 vs 13,422 pg mL^-1^ at T0 and T4, respectively). If the co-culture of Caco-2 and THP-1 was first established and the THP-1 cells exposed to LPS at T0 (B, middle), the release of IL-1β and TNF-α was nearly inhibited. The levels of IL-8 were markedly reduced by 33% compared to the T4 results from co-cultures with LPS pre-exposed THP-1 cells. (mean of n=1 with three technical replicates of 1 experiment performed)





**Fig. S8. TEER over 48h inflamed model co-culture with and without supernatant replacement for LY barrier crossing studies:** To study the Caco-2 barrier’s apparent permeability to LY in the inflamed model co-culture two different approaches of incubation with LY were tried. In the first approach (T_24_, squares), the inflamed model co-culture was established as described before and maintained for 24h. After 24h, the supernatant was replaced with HBSS (containing Mg^+^ and Ca^2+^) in the BL and HBSS + LY in the AP compartment. After the exchange of supernatant (red squares) the TEER quickly re-established to 110% and 150% of the monoculture control after 4 and 24h, respectively. Using the second approach, the inflamed model co-culture was established with LY dissolved in the apical CCM and maintained for 18h (green triangles). The TEER decreased similarly to the inflamed control co-culture in presence of LY. (mean ± s.d. of n=1 with three technical replicates of 1 experiment performed)





**Fig. S9. Apparent permeability (P_app_) of Caco-2 barriers to Lucifer Yellow after 4h (T_24_) or 18h (T_0_) Caco-2 monoculture, stable or inflamed model co-culture**: To detect an increase in barrier leakage in the inflamed model the P_app_ to LY was quantified using two different approaches: in approach 1 (T_0_) LY was dissolved in the CCM of the AP compartment and the cultures established as described before. After 18h of culture, samples were taken from the AP and BL compartment of all three conditions to calculate the P_app_. In the second approach (T_24_), the stable and inflamed conditions were established and maintained for 24h before LY dissolved in HBSS was added to the AP compartment. Samples for the calculation the P_app_ were taken 4h after the addition of LY. In neither of the approaches the P_app_ for LY was increased in the inflamed condition compared to the Caco-2 monoculture and stable co-culture. (mean ± s.d. of n=1 with 3 technical replicates of 1 experiment performed; LY added: T24 Inflamed: n=2)


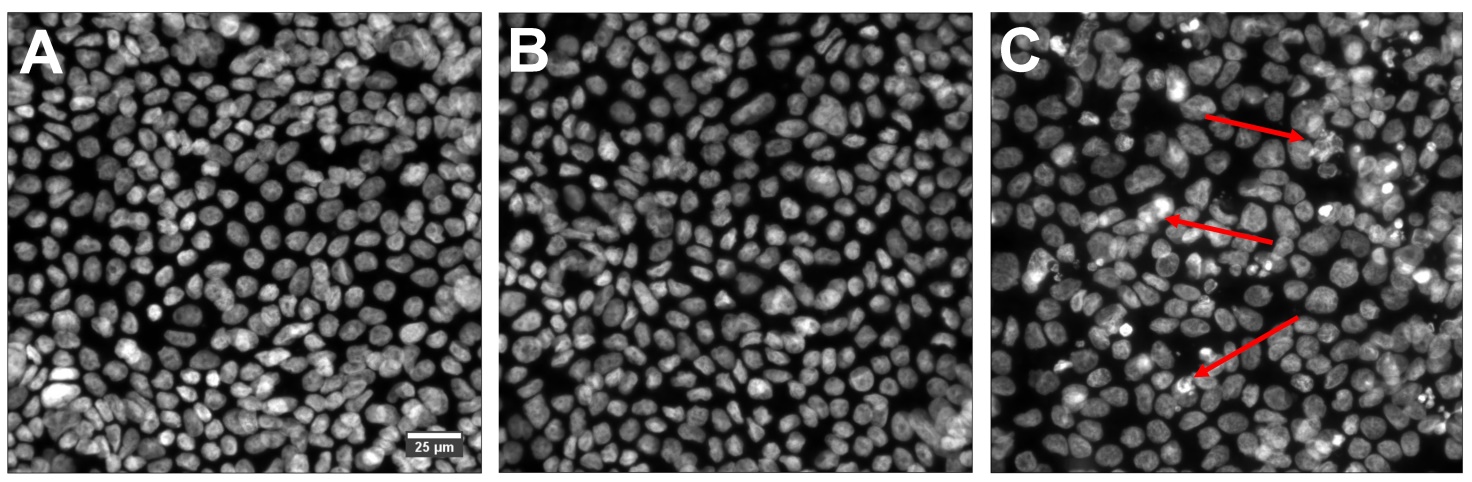


**Fig. S10. Nuclear integrity of the Caco-2 cell layer in monoculture or after 48h of stable or inflamed co-culture**: The images show no clear differences in the Caco-2 monolayer integrity between the Caco-2 monoculture (A) and stable co-culture (B). In both conditions, the cells are evenly distributed and form a dense and continuous layer. The Caco-2 cell layer of the inflamed co-culture (C) shows a striking occurrence of fragmented and condensed nuclei (red arrows).

| **A** |  |
| --- | --- |
| **B** |  |

**Fig. S11. Cytokine release after 28h Caco-2 monoculture, stable, or inflamed co-culture:** (A) TNF-α, IFN-γ, IL-4, IL-6, MCP-1 and GM-CSF expressed in pg mL^-1^, (B) MIP-1α expressed as measured FI (mean ± s.d.; n=6; *p≤0.05 compared to monoculture, ^#^p≤0.05 compared to stable co-culture)


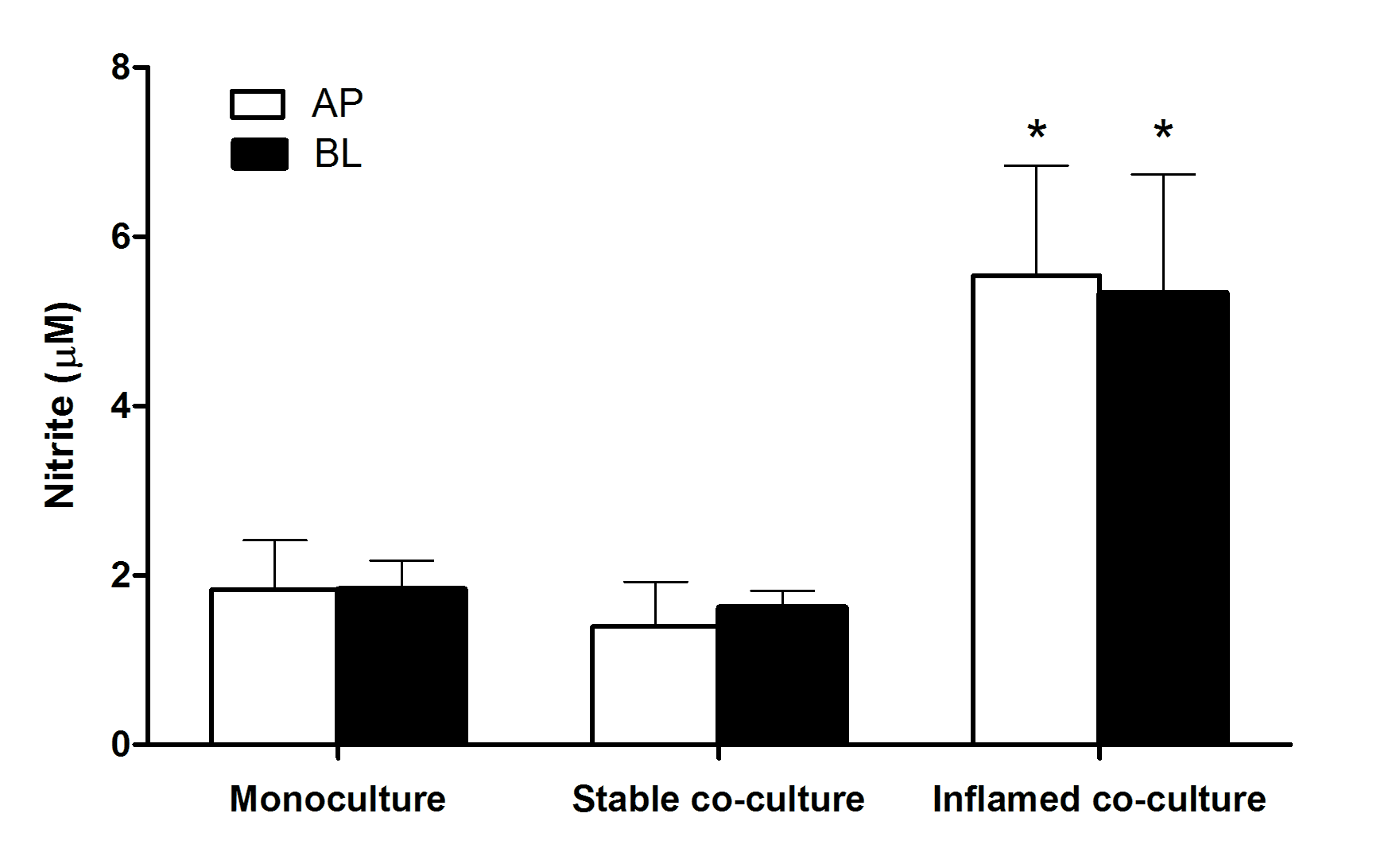


**Fig. S12. NO_2_^-^ quantification after 48h Caco-2 monoculture, stable and inflamed model co-culture:** In both Caco-2 monoculture and stable co-culture very low concentrations of NO_2_^-^ were detected. In the inflamed model the amount of NO_2_^-^ was clearly increased to ~6 μM in AP and BL compartment. It cannot be said with certainty whether the NO_2_^-^ was released from both or only one cell line. (mean ± s.d.; *p≤0.05 compared to NO release in Caco-2 monoculture and stable co-culture)





**Fig. S13. TEER over 48h stable co-culture with exposure to EDTA and LPS:** The apical and basolateral exposure to 2.5 mM EDTA caused a rapid and strong reduction in TEER. After 4h of exposure, the TEER reduced to ~20% of the Caco-2 monoculture control and did not re-establish again over the following 44h of culture. The persistent TEER reduction suggests a significant and permanent barrier disruption through the disintegration of the Caco-2 cell layer. (mean ± s.d., T18: n=2)

| **A** |  |
| --- | --- |
| **B** |  |

**Fig. S14. TEER over 48h (A) Caco-2 monoculture with AP and / or BL exposure to LPS and (B) stable co-culture with AP exposure to LPS or LPS +IFN-γ** (mean ± s.d.; (A) Monoculture LPS (AP) and LPS (AP+BL) n=2; (B) stable co-culture: n=1 with three replicates of 1 experiment performed)

| **A** |  |
| --- | --- |
| **B** |  |

**Fig. S15 Cytokine response after 24h and 48h: Caco-2 monoculture exposed to EDTA+LPS, THP-1 monoculture exposed to EDTA, stable co-culture apically exposed to LPS (AP) and stable co-culture apically exposed to EDTA, LPS, and IFN-γ:** The exposure of Caco-2 monocultures to EDTA and LPS for (A) 24h and (B) 48h did not result in increased release of IL-1β, IL-8 or TNF-α. No unusually high release of pro-inflammatory cytokines was detected in EDTA-exposed THP-1 monocultures or apically LPS-exposed stable co-cultures without addition of EDTA. In comparison to the stable co-culture apically treated with EDTA and LPS (Fig. 8) the additional presence of IFN-γ induced a significantly increased release of all three pro-inflammatory cytokines in the stable co-culture. (mean ± s.d. of n=1 with 3 technical replicates of 1 experiment performed)
